# Reducing antibiotic treatment duration for ventilator-associated pneumonia (REGARD-VAP): a trial protocol for a randomised clinical trial

**DOI:** 10.1136/bmjopen-2021-050105

**Published:** 2021-05-13

**Authors:** Yin Mo, Timothy Eoin West, Graeme MacLaren, Suchart Booraphun, Andrew Yunkai Li, Gyan Kayastha, Yie Hui Lau, Yin Tze Chew, Ploenchan Chetchotisakd, Paul Anantharajah Tambyah, Direk Limmathurotsakul, Ben Cooper

**Affiliations:** 1 Centre for Tropical Medicine and Global Health, Nuffield Department of Medicine, University of Oxford, Oxford, UK; 2 Mahidol-Oxford Tropical Medicine Research Unit, Faculty of Tropical Medicine, Mahidol University, Bangkok, Thailand; 3 University Medicine Cluster, National University Hospital, Singapore; 4 Department of Medicine, National University of Singapore, Singapore; 5 International Respiratory and Severe Illness Center, University of Washington, Seattle, Washington, USA; 6 Division of Pulmonary and Critical Care Medicine, Department of Medicine, University of Washington, Seattle, Washington, USA; 7 National University Heart Centre, National University Hospital, Singapore; 8 Medical Department, Sunpasithiprasong Hospital, Ubon Ratchathani, Thailand; 9 Patan Hospital, Patan Academy of Health Sciences, Kathmandu, Nepal; 10 Anaesthesiology, Intensive Care and Pain Medicine, Tan Tock Seng Hospital, Singapore; 11 Department of Medicine, Srinagarind Hospital, Faculty of Medicine and Research and Diagnostic Center for Emerging Infectious Diseases (RCEID), Khon Kaen University, Khon Kaen, Thailand; 12 Infectious Diseases Translational Research Programme, Department of Medicine, Yong Loo Lin School of Medicine, National University of Singapore, Singapore

**Keywords:** infectious diseases, intensive & critical care, statistics & research methods

## Abstract

**Introduction:**

Ventilator-associated pneumonia (VAP) is the most common nosocomial infection in intensive care units (ICUs). Using short-course antibiotics to treat VAP caused by Gram-negative non-fermenting bacteria has been reported to be associated with excess pneumonia recurrences. The “REducinG Antibiotic tReatment Duration for Ventilator-Associated Pneumonia” (REGARD-VAP) trial aims to provide evidence for using a set of reproducible clinical criteria to shorten antibiotic duration for individualised treatment duration of VAP.

**Methods and analysis:**

This is a randomised controlled hierarchical non-inferiority–superiority trial being conducted in ICUs across Nepal, Thailand and Singapore. The primary outcome is a composite endpoint of death and pneumonia recurrence at day 60. Secondary outcomes include ventilator-associated events, multidrug-resistant organism infection or colonisation, total duration of antibiotic exposure, mechanical ventilation and hospitalisation. Adult patients who satisfy the US Centers for Disease Control and Prevention National Healthcare Safety Network VAP diagnostic criteria are enrolled. Participants are assessed daily until fever subsides for >48 hours and have stable blood pressure, then randomised to a short duration treatment strategy or a standard-of-care duration arm. Antibiotics may be stopped as early as day 3 if respiratory cultures are negative, and day 5 if respiratory cultures are positive in the short-course arm. Participants receiving standard-of-care will receive antibiotics for at least 8 days. Study participants are followed for 60 days after enrolment. An estimated 460 patients will be required to achieve 80% power to determine non-inferiority with a margin of 12%. All outcomes are compared by absolute risk differences. The conclusion of non-inferiority, and subsequently superiority, will be based on unadjusted and adjusted analyses in both the intention-to-treat and per-protocol populations.

**Ethics and dissemination:**

The study has received approvals from the Oxford Tropical Research Ethics Committee and the respective study sites. Results will be disseminated to patients, their caregivers, physicians, the funders, the critical care societies and other researchers.

**Trial registration number:**

NCT03382548.

**Strengths and limitations of this study**

- The “REducinG Antibiotic tReatment Duration for Ventilator-Associated Pneumonia” (REGARD-VAP) trial is a randomised controlled hierarchical non-inferiority–superiority trial which compares a short duration treatment strategy versus a standard-of-care duration for ventilator-associated pneumonia (VAP).
- The short treatment strategy allows for individualisation of antibiotic duration recommendations according to the patients’ clinical responses, that is, antibiotics can be stopped after 48 hours of defervescence and stable haemodynamics parameters.
- The trial will update treatment duration guidelines for VAP predominantly caused by Gram-negative non-fermenting bacilli, which were previously reported to be associated with more frequent recurrences.
- To overcome the anticipated issue of non-adherence to allocated treatment, which potentially may increase type 1 error and bias the study estimates, multiple analysis approaches will be performed in both intention-to-treat and per-protocol populations including inverse probability weighting.
- The REGARD-VAP trial excludes patients with concurrent infections from other sources and who are immunocompromised.

## Introduction

Ventilator-associated pneumonia (VAP) is the most common hospital-acquired infection in patients admitted to the intensive care unit (ICU).^1^ Estimates of all-cause mortality in patients with VAP range from 20% to 50%,^2 3^ and can be as high as 94% in low-income and middle-income countries.^4^ Given its high prevalence and frequent association with multidrug-resistant organisms, the treatment of VAP is likely to be a key driver of antimicrobial resistance (AMR) in ICUs.

We continue to rely on clinical, radiographical and microbiological criteria with low sensitivity and specificity (~70 and ~75%, respectively) to diagnose VAP.^5^ Identification of causative organisms can be difficult as the upper respiratory tract is non-sterile and can contaminate specimen collection from the lower respiratory tract. Concordance between tracheal non-quantitative cultures and cultures of lung tissue from open lung biopsy has been found to be as low as 40%.^6^ These factors result in overdiagnosis and overtreatment of organisms thought to be causing VAP with empirical combinations of broad-spectrum antibiotics.

For those patients who are prescribed culture-directed definitive antibiotics, duration of treatment remains controversial. There are two notable French clinical trials that have suggested that a short course of 7–8 days has comparable clinical efficacy as a long duration of 15 days.^7 8^ However, these studies could not confidently conclude that the finding can be applied to VAP caused by Gram-negative non-fermenting (GNNF) bacilli due to increased recurrence in such patients (OR 2.18; 95% CI 1.14 to 4.16).^7^ Important potential biases exist in these studies, for example due to the differential time period during which recurrence was assessed and the potential for erroneous classification of persistent colonisation as recurrent infection. Moreover unplanned subgroup analyses are known to be unreliable and the higher rate of recurrence in pneumonias caused by GNNF bacilli could simply be a chance association.^7 8^

Furthermore, the chosen empirical duration of 7 days in the above trials did not make use of individual patients’ clinical responses to guide antibiotic duration. Some studies have suggested treatment duration less than 7 days suffice for VAP. One conducted by Singh *et al*^9^ evaluated 3 days of empirical ciprofloxacin monotherapy for patients who satisfy a set of clinical criteria signifying low likelihood of active VAP at day 3 of treatment. Compared with those who received longer duration of antibiotics, there was no difference in mortality or ICU length of stay. Another randomised study by Micek *et al*^10^ adopted an antibiotic discontinuation policy to shorten VAP treatment. Similarly, there was no difference between the short (6.0±4.9 days) and long duration treatment groups in terms of mortality and VAP recurrences. The above evidence supports an individualised duration of antibiotic treatment for VAP depending on disease severity and clinical response.

Another gap in the evidence is that there is no randomised study defining antibiotic treatment duration for culture-negative VAPs. There are two observational studies that compared outcomes between those whose antibiotics were withheld on the basis of negative respiratory cultures and those whose antibiotics were continued.^11 12^ Patients whose antibiotics were discontinued did not have a higher mortality or rate of new respiratory infection compared with patients whose antibiotics were continued.

The above limits the applicability of the current recommendation for short-course antibiotics for VAP, especially in Asia where most of these infections are caused by GNNF bacilli such as *Pseudomonas aeruginosa* and *Acinetobacter baumannii*.^13^ The current median number of days of antibiotic treatment remains at 12–13 days in the academic centres in Thailand.^14 15^ We present a VAP trial protocol addressing the above issues, which compares the clinical outcomes of short-course antibiotic treatment strategy versus standard-of-care duration in adult patients with VAP, with the aim of reducing unnecessary antibiotic use and AMR selective pressure in the ICUs.

## Methods and analysis

### Study design

The REGARD-VAP trial is a multicentre randomised controlled hierarchical non-inferiority–superiority trial to assess the clinical effect of a short versus standard-of-care duration in adults with VAP. The short-course treatment strategy considers the participants’ clinical response, defined by defervescence for 48 hours and stabilising blood pressure and discontinues antibiotics within 7 days of treatment.

### Institutional review board approval

The overall sponsor of the study is the University of Oxford. The Oxford Tropical Research Ethics Committee approval was obtained prior to applications to the respective local ethics committees. Written informed consent is obtained from every participant, or the participant’s legal representative or next-of-kin if the participant is sedated and does not have decision-making capacity. When the participant is deemed to have decision-making capacity, he/she is reconsented. This trial protocol was developed in accordance with the SPIRIT 2013 Statement and Consolidated Standards of Reporting Trials(CONSORT) statement extension for ‘Non-inferiority and Equivalence Trials’.^16 17^ The full protocol is provided in online supplemental file 1.

10.1136/bmjopen-2021-050105.supp1 Supplementary data

### Eligibility criteria

We adopt the US Centers for Disease Control and Prevention (CDC) National Healthcare Safety Network (NHSN) VAP diagnostic criteria on patients who have been mechanically ventilated for ≥48 hours as our study subject inclusion criteria.^18^ While we acknowledge that there are no ‘gold standard’ diagnostic criteria for VAP and that clinical criteria correlate poorly with autopsy findings (previously determined to be 69% sensitivity and 75% specificity), the CDC NHSN diagnostic criteria are sensitive and practical for use in ICUs of various resources and settings.^19^ Only one episode of suspected VAP per participant is included.

We agree with the 2016 Clinical Practice Guidelines by the Infectious Diseases Society of America (IDSA) and the American Thoracic Society (ATS) that the additional use of infection scores and other biomarkers including procalcitonin, C-reactive protein and soluble triggering receptor expressed on myeloid cells-1 lack sensitivity and specificity.^2^ For these reasons, they are not included in our inclusion criteria. Microbiological culture results are not part of our inclusion criteria so that we are able to recruit patients who have suspected VAP but respiratory cultures are negative. The specific inclusion and exclusion criteria are as follows.

Inclusion criteria:

- Patients 18 years and older.
- Invasive mechanical ventilation ≥48 hours.
- At least one of the following:
  1. temperature >38°C.
  2. white cell count ≥12 ×10^9^/L or ≤4 ×10^9^/L.
  3. altered mental status with no other causes in >70 year olds; AND
- Two or more chest imaging tests demonstrating at least one of the following:
  1. new and progressive OR progressive and persistent infiltrate.
  2. new and persistent OR progressive and persistent consolidation.
  3. new and persistent OR progressive and persistent cavitation, AND
- At least two of the following:
  1. new onset of purulent sputum, or change in character of sputum, or increased respiratory secretions or increased in suctioning requirements.
  2. new onset or worsening tachypnea or dyspnoea.
  3. rales or bronchial breath sounds.
  4. worsening gas exchange defined by oxygen desaturations (eg, PaO_2_/FiO_2_<240), increased oxygen requirements or increased ventilation demand.

Exclusion criteria:

- Poor likelihood of survival as defined by a Sepsis-related Organ Failure Assessment score (SOFA score) of >11 points.^20^
- Immunocompromised patients (HIV with CD4 <200 cells/mm^3^, corticosteroids > 0.5 mg/kg per day for >30 days, received chemotherapy in the past 3 months, solid organ or haematopoietic cell transplant).
- Patients receiving antibiotic therapy for any other defined extra-pulmonary infections that warrant a duration of antibiotics longer than 7 days or complications of pneumonia such as lung abscess or empyema, that warrant a duration of antibiotics longer than 7 days (excluding antituberculosis treatment, antifungal medications, antibiotics meant for chronic suppression of chronic infections or chronic obstructive lung disease).
- Patients who have been treated for VAP for more than 7 days from screening.
- Vulnerable patients including prisoners and refugees.

### Recruitment and participating sites

The trial is conducted in ICUs across Singapore, Thailand and Nepal. These hospitals include university academic and provincial-level centres from various resource settings to ensure generalisability of the trial findings. Enrolment to the study commenced in June 2018. As of November 2020, there are 31 participating ICUs from 5 hospitals. These hospitals are:

1. National University Hospital, Singapore.
2. Tan Tock Seng Hospital, Singapore.
3. Sunpasitthiprasong Hospital, Ubon Ratchathani, Thailand.
4. Srinagarind Hospital, Khon Kaen, Thailand.
5. Patan Hospital, Patan Academy of Health Science, Nepal.

Dedicated research teams in each study site screen all admitted patients who have been intubated for more than 48 hours for eligibility. Potential participants are then recruited by the local investigators.

### Randomisation and blinding

Randomisation is done in a 1:1 ratio, via permuted blocks and stratified by study sites. The randomisation sequence is generated with a computer programme using a seed to allow reproducibility. Allocation is performed using sequentially numbered opaque envelopes. Fitness criteria to discontinue antibiotics must be met prior to randomisation.

Patients are blinded as they are not informed of the treatment duration and likely to be sedated and unaware of the treatment regimens. To minimise observer bias by the primary physicians and study investigators, randomisation takes place after study participants have met the fitness criteria, such that study participants do not receive differential treatments during the episode of VAP except from antibiotic duration. After randomisation, investigators will contact the primary physicians to stop antibiotics for those participants randomised to the short treatment arm. Independent experts, who are assigned to determine pneumonia recurrences, will be blinded to the randomisation arms.

### Baseline procedures

On enrolment, a set of respiratory cultures is performed if this has not been done for the index episode of VAP. This may be collected either via the endotracheal tube or bronchoalveolar lavage as ordered by the primary physicians. Brocheoalveolar lavage is not mandated as this has been shown not to improve mortality and clinical outcomes and may be difficult to perform in low-resource settings.^21 22^ Microbiology cultures are processed and reported in the local laboratories which are expected to have standing quality control measures. Susceptibility studies are reported using European Committee on Antimicrobial Susceptibility Testing or Clinical and Laboratory Standards Institute agar method and breakpoints.^23 24^

Relevant clinical and laboratory-related information including demographics, medical history, antibiotics administration record, chest X-ray or other imaging findings, biochemical, microbiological and haematological results and clinical parameters are collected using the case record form (CRF).

### Treatment protocols and intervention

**Figure 1:**
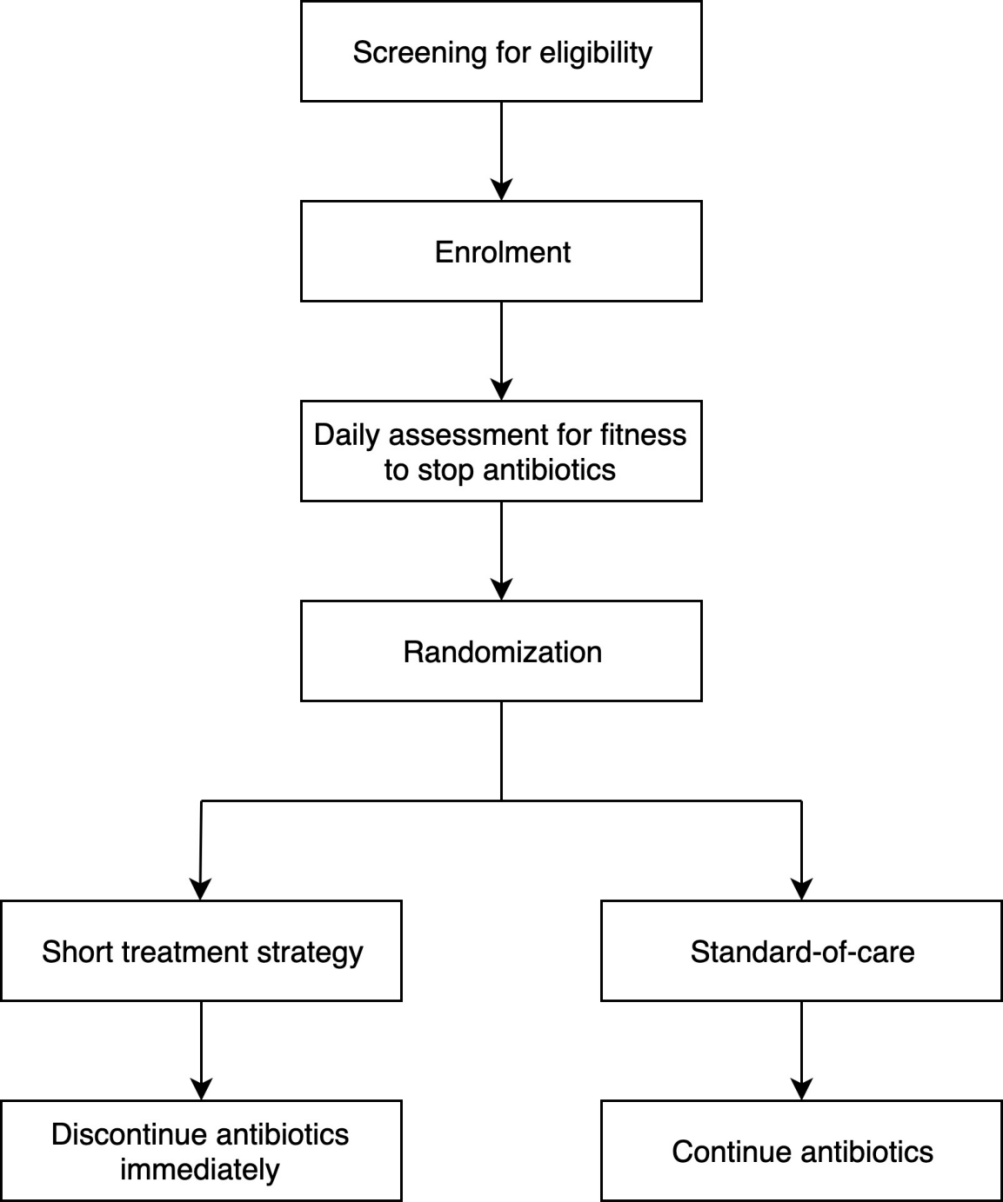
Study treatment protocol flow diagram.

Antibiotic treatment for VAP is tailored to the susceptibilities of the respiratory pathogens in accordance with the 2016 IDSA/ATS VAP guideline.^2^ The number of days of antibiotics is calculated from the first day of appropriate coverage according to the susceptibility of at least one of the pathogens recovered from respiratory cultures taken within 48 hours of screening or VAP symptom onset. Primary physicians are encouraged to convert initial empirical regimen to narrow-spectrum therapy based on culture results. In culture-negative cases, empirical antibiotic choice is made depending on local hospital antibiograms reported by the respective microbiology laboratories (figure 1).

Following enrolment, participants are reviewed daily for fitness criteria to stop antibiotics (table 1). These criteria include: (a) body temperature ≤38.3°C (core body temperature measured orally or rectally) or 38°C (axillary) for 48 hours, and (b) haemodynamic instability (systolic blood pressure ≥90 mm Hg without inotropic support or no requirement of inotropic support to maintain systolic blood pressure above 90 mm Hg).

**Table 1:** Standard Protocol Items: Recommendations for Interventional Trials (SPIRIT) figure of the schedule of enrolment, interventions and assessments

Timepoint	Study period				
	Enrolment	Allocation	Post-allocation		Close-out
	Day 0	Days 0–7	Daily/ weekly during hospitalisation*	Day 28	Day 60
Enrolment					
Eligibility screen	X				
Informed consent	X				
Sputum culture	X				
Assessment					
Vitals and medical chart review	X	X	X	X	X
Antibiotics review	X	X	X	X	X
Allocation		X			
Sputum and stool sample collection	X		X	X	X
Recurrence review			X	X	X
Intervention					
Discontinuation of antibiotic		X			

*Participants will be followed up daily while on antibiotics, and then weekly until discharge.

When the above criteria are met, all antibiotics for participants randomised to the short duration treatment strategy arm are stopped as early as day 3 if the respiratory culture is negative, or day 5 if the respiratory culture is positive. Antibiotics administered via all routes that is, intravenous, oral and nebulisation should be stopped within 7 days. Participants in the standard-of-care arm receive antibiotic treatment for at least 8 days with the exact duration decided by the primary physician.

### Strategies to ensure adherence and assessment of adherence to protocol

Non-adherence, especially in non-inferiority trials, is challenging to account for in the analysis and complicates interpretation of results.^25–27^ To maintain engagement with the local investigators and healthcare providers, the study team carries out regular meetings with the stakeholders to elicit feedback on study procedures. Prior to enrolment and randomisation, the study team contacts the primary physicians to confirm their adherence to allocated interventions. Post-randomisation, close follow-ups are done to ensure antibiotics are stopped or continued according to allocation.

Adherence is assessed by duration of culture-directed antibiotics. A participant is considered to meet the definition of per-protocol if he/she fulfilled the eligibility criteria for enrolment, fitness criteria for discontinuation of antibiotics and received 7 days or fewer of appropriate antibiotics in the short treatment strategy arm, and 8 days or more of appropriate antibiotics in the standard-of-care arm.

### Follow-up

Participants are followed-up daily while on antibiotics, and subsequently weekly when remaining hospitalised. Following discharge, two further follow-ups are scheduled at days 28 and 60 (table 1). During follow-up visits, patients are interviewed to identify possible episodes of pneumonia recurrences.

### Outcomes and measures

The primary outcome is the composite endpoint of death and pneumonia recurrence within 60 days of enrolment. Recurrent pneumonia is defined as an additional episode of pneumonia as determined by two independent infectious disease or respiratory medicine experts blinded to the randomisation. Day 60 is chosen for the primary outcome in preference to day 30 to reduce bias that may occur with participants in the short treatment strategy arm having more antibiotic-free days, thereby leading to a differential detection of recurrences between the arms. Previous observational studies suggest that mortality attributable to VAP persists to day 60.^28^

The secondary outcomes are ventilator-associated events, duration of mechanical ventilation, duration of hospitalisation, duration of exposure to antibiotics during hospitalisation, acquisition of multidrug-resistant infection or colonisation during hospitalisation, and the number and types of extra-pulmonary infections identified from sterile sites during hospitalisation.

Detailed definitions of the above outcomes are provided in online supplemental file 2. For all these outcomes, we will calculate the absolute risk difference between the proportion of participants with the outcomes in the standard-of-care arm and the short treatment strategy arm.

10.1136/bmjopen-2021-050105.supp2 Supplementary data

### Statistical analysis plan

The primary and secondary outcomes of the study populations will be analysed using unadjusted and adjusted methods in both the per-protocol and intention-to-treat populations. The per-protocol population includes all study participants who fulfil the eligibility criteria, undergo randomisation, meet fitness criteria for antibiotic discontinuation, and who receive 7 days or fewer of appropriate antibiotics in the short treatment strategy arm and 8 days or more in the standard-of-care arm. The intention-to-treat population includes all study participants who have been randomised during the conduct of the study. Adjustment will be done with inverse probability weighting, using baseline patient characteristics (study site, age, gender, comorbidities, residence prior to admission, type of ICU admitted to, SOFA score, VAP infection with carbapenem-resistant organisms, maximum heart rate and minimum mean arterial blood pressure on randomisation day, duration of intubation prior to developing VAP, reason for intubation) as independent variables.^29^ These are potential confounders that are not on the direct causal pathway between duration of antibiotic treatment and the outcomes.^30^

This trial has a hierarchical non-inferiority–superiority hypothesis. The first analysis to be conducted will be for determination of non-inferiority. Only if non-inferiority is established by this primary analysis, will a second analysis for superiority be conducted using closed testing methods without requiring adjustment of the significance level for multiple comparisons.^25^ Non-inferiority will be concluded if the upper limit of the one-sided 95% confidence intervals from both adjusted analyses do not cross the non-inferiority margin. The purpose of using both adjusted analyses on the intention-to-treat and per-protocol populations to determine non-inferiority is to minimise the inflation of type 1 error associated with non-adherence in non-inferiority trials.^17 26 27 31^ Superiority will be declared if the entire confidence intervals for all the trial estimates are below zero.

### Sample size calculation and non-inferiority margin determination

Mortality after sustaining an episode of VAP has been reported to be 14%–43% globally.^8 32–39^ VAP recurrence rates range from 14% to 40%, with higher incidence in those caused by GNNF bacilli.^33 40 41^ Mortality observed in these recurrence episodes were 17%–50%.^32 33^ Based on these, we expect the primary outcome (a composite binary outcome of mortality and VAP recurrence) to occur in 55% of the patients in the standard-of-care arm. We derived an absolute non-inferiority margin of 12% with the fixed-margin method, preserving at least 50% of the efficacy of standard treatment in VAP.^42 43^ Using a group sequential design adopting the boundaries proposed by Fleming-Harrington-O’Brien, a maximum of 412 patients will be required to achieve a power of 80% to conclude non-inferiority between the two groups with a one-sided α risk of 5%.^44^ As we anticipate a loss to follow-up of up to 10%, we plan to enrol a maximum of 460 patients.

### Data collection and management

Paper CRFs are completed at the study sites and entered onto an electronic database, MACRO Electronic Data Capture.^45^ A dedicated data manager and study monitor at the Mahidol-Oxford Research Unit Clinical Trial Support Team supervise the overall quality of the data collection. The data manager reviews data entered on a monthly basis and any unexpected values are clarified with the site study teams. The final dataset will be archived in the data repository after the publication.

The study monitor and a project coordinator conduct regular sites visits for quality control. All study sites are assessed prior to initiation of the study for capacity to conduct the randomised controlled trial, during the study and on completion to ensure data quality. Monitoring reports are made available to the study sites and investigators after each visit.

### Safety monitoring plan

Serious adverse events, including all mortality and pneumonia recurrences, are reported to the local ethics committees and study sponsor according to the respective requirements and timeline. The data safety and monitoring committee (DSMC) consisting of an infectious disease physician, a respiratory/intensive care physician and a statistician, is responsible for the monitoring and evaluation of the clinical data generated, with a focus on safety in an independent and objective manner. The DSMC reviews all serious adverse events on a monthly basis, and interim analysis reports to make recommendations on study conduct such as continuation, modification, suspension and termination. A trial steering committee will also be constituted and will decide on the continuation of the trial and will report to the central ethics committee.

Four interim analyses will be performed on the primary endpoint whenever 25% of the estimated sample size has been randomised and have completed 60 days of follow-up. We will use the group sequential design adopting the boundaries proposed by Fleming-Harrington-O’Brien to terminate the trial prematurely once the Z value derived exceeds the defined boundaries for superiority.^44 46 47^ (table 2)

**Table 2:** α-error spending and power at each interim analysis for determination of non-inferiority

Interim analysis	Z-value upper bound	α-error spending	Power
1	3.47	0.00026	0.014
2	2.45	0.00697	0.241
3	2.00	0.01795	0.329
4	1.73	0.02482	0.216
Total		0.05	0.80

### Qualitative component and implementation of intervention

The study team engaged with the local investigators and ICU healthcare providers during the trial design stage and subsequently while conducting the study to gather feedback on the acceptance of the study intervention and anticipate practical and operational issues. An important debate was on the indicators of fitness to stop criteria. There were suggestions to include inflammatory markers such as procalcitonin as criteria to discontinue antibiotics. This approach has previously been shown to reduce antibiotic use in patients without increase in adverse events.^48 49^ However, as such tests are not routinely available in Thailand and Nepal, and performing these tests offsite would be likely to cause prolonged delay in discontinuation of antibiotics, a decision was made not to include these as part of our intervention.

Early discontinuation of antibiotics was also deemed to be a challenge to implement for some intensive care physicians due to variations in practices. Champions for the trial were identified locally, who advocated for the trial intervention prior to initiation. External pilots were conducted when required, for the sites to improve adherence in the main trial.^50^

A qualitative study is being performed concurrently at the study sites to understand the interactions of the ICU staff with the trial procedures with the aims of improving adherence to the study protocol and implementing the intervention as part of local antibiotic stewardship programmes if the trial demonstrates non-inferiority.

### Laboratory studies

Respiratory and stool samples are collected from the study participants weekly during hospitalisation, then at days 28 and 60. These samples will undergo DNA extraction and shotgun metagenomics analysis. The characteristics of the microbiota will be compared between the groups of patients to explore the short-term and long-term impact of the various regimes and durations of antibiotics.

### Patient and public involvement

Patients and the public were not involved in the design of the study.

### Ethics and dissemination

The REGARD-VAP trial has been approved by the following ethics committees: Oxford Tropical Research Ethics Committee (40-17), Srinagarind Hospital Center for Ethics in Human Research (4.6.03: 59/2563), Sunpasitthiprasong hospital Center for Ethics in Human Research (085/2561), Singapore National Health Group Domain Specific Review Board (2018/00250) and Nepal Health Research Council (630-2018). The trial results will be disseminated to patients, their caregivers, physicians, the funders, the critical care societies and other researchers.

## Supplementary Material

Reviewer comments
