# Supplementary material for: Reducing antibiotic treatment duration for ventilator-associated pneumonia (REGARD-VAP): a trial protocol for a randomised clinical trial
Source: BMJ Open. 2021 May 13;11(5):e050105. doi: 10.1136/bmjopen-2021-050105 (PMC8126270; doi:10.1136/bmjopen-2021-050105)
Supplement: Supplementary data [file bmjopen-2021-050105supp001.pdf]

Study Title: **RE**ducin**G** **A**ntibiotics t**R**eatment **D**uration for **V**entilator-**A**ssociated **P**neumonia (REGARD-VAP)

Protocol Number : BAC17008

Trial registration number : NCT03382548

OxTREC Ref: 40-17

Date and Version No: version. 4.0 dated 15 October 2019

**Chief Investigator:**	Ben Cooper<br>Mahidol-Oxford Tropical Medicine Research Unit, Mahidol University and University of Oxford
**Investigators:**	Direk Limmathurotsakul<br>Mahidol-Oxford Tropical Medicine Research Unit, Mahidol University and University of Oxford
	Mo Yin<br>National University Health System, Singapore and University of Oxford
	Paul Tambyah<br>National University of Singapore
**Site investigators:**	Andrew Li<br>National University Health System, Singapore
	Lau Yie Hui<br>Tan Tock Seng Hospital, Singapore
	Gyan Kayastha<br>Patan Hospital, Patan Academy of Health Sciences, Nepal
	Suchart Booraphun<br>Sunpasithiprasong Hospital, Thailand
	Ploenchan Chetchotisakd, Professor<br>Srinagarind Hospital, Thailand
**Sponsor:**	University of Oxford<br>Centre for Tropical Medicine, Nuffield Department of Medicine, University of Oxford, Old Road Campus, Headington, Oxford, United Kingdom, OX3 7BN

Mo Y, *et al. BMJ Open* 2021; 11:e050105. doi: 10.1136/bmjopen-2021-050105

Funders: The project is jointly funded by Medical Research Council/ Department for International Development (MRC/DfID) (Grant Ref: MR/K006924/1), and Singapore National Medical Research Council (Grant ref: CTGIIT18may-0003).

Potential conflicts of interest:

There are no conflicts of interest to declare. The study sponsor and funders will have no role in data collection, management, analysis, and interpretation of data; writing of the report; and will not hold the decision to submit the report for publication.

TABLE OF CONTENTS

1. SYNOPSIS 5
2. ABBREVIATIONS 6
3. BACKGROUND AND RATIONALE 7
4. OBJECTIVES AND OUTCOME MEASURES 9
5. STUDY DESIGN 10
6. PARTICIPANT IDENTIFICATION AND RECRUITMENT 13
6.1. Study Participants 13
6.2. Inclusion Criteria 13
6.3. Exclusion Criteria 13
7. STUDY PROCEDURES 13
7.1. Recruitment 13
7.2. Screening and Eligibility Assessment 14
7.3. Informed Consent 14
7.4. Randomisation, blinding and unblinding 15
7.5. Baseline Assessments 15
7.6. Subsequent Visits 15
7.7. Sample Handling 15
7.8. Discontinuation/Withdrawal of Participants from Study 17
7.9. Definition of End of Study 17
8. INTERVENTIONS / INVESTIGATIONS 17
9. SAFETY REPORTING 18
9.1. Definition of Serious Adverse Events 18
9.2. Reporting Procedures for Serious Adverse Events 19
10. STATISTICS AND ANALYSIS 19
10.1. Description of Statistical Methods for Primary and Secondary Outcomes 19
10.2. The Number of Participants 20
10.3. Analysis of microbiota and whole genome sequencing 20
11. DATA MANAGEMENT 21
11.1. Access to Data 21
11.2. Data Handling and Record Keeping 21
12. QUALITY CONTROL AND QUALITY ASSURANCE PROCEDURES 21
13. ETHICAL AND REGULATORY CONSIDERATIONS 22
13.1. Declaration of Helsinki 22

13.2. Guidelines for Good Clinical Practice 22
13.3. Approvals 22
13.4. Participant Confidentiality 22
13.5. Expenses and Benefits 22
13.6. Reporting 22
14. FINANCE AND INSURANCE 22
14.1. Funding 22
14.2. Insurance 23
15. PUBLICATION POLICY 23
16. REFERENCES 24
17. APPENDIX A: STUDY FLOW CHART 27
18. APPENDIX B: SCHEDULE OF STUDY PROCEDURES 29

Protocol version 4.0 dated 15 October 2019

1. SYNOPSIS

**Study Title**	**RE**ducin**G** **A**ntibiotics t**R**eatment **D**uration for **V**entilator-**A**ssociated **P**neumonia (REGARD-VAP)	
**Protocol no.**	BAC17008	
**Study Design**	Randomised, partially double-blind (blinding may be achieved up to 7 days) controlled trial to assess the noninferiority of a short duration of antibiotics (up to 7 days) versus prolonged antibiotic therapy (as per physician preference) in adults patients with VAP in Asia	
**Study Participants**	Adult patients (≥18 years old) who satisfy inclusion and exclusion criteria for VAP	
**Planned Sample Size**	460	
**Planned Study Period**	2018 January – 2021 June	
	**Objectives**	**Outcome Measures**
**Primary**	To demonstrate clinical non-inferiority of a shorter antibiotic treatment duration compared to a longer duration in adult patients with VAP	Difference in the proportion of participants with the composite endpoint of death and VAP recurrence rate within 60(±5) days of enrolment
**Secondary**	To define other clinical benefits of shorter antibiotic treatment duration compared to a longer duration in adult patients with VAP	Ventilator-associated events, duration of mechanical ventilation, duration of hospitalisation, acquisition of multidrug resistant infection or colonisation during the hospitalisation, number of days of exposure to antibiotics during the hospitalisation, number and types of extrapulmonary infections during hospitalisation (determined from cultures taken from sterile sites)
	To study the impact of various antibiotic regimen on the microbial community dynamics and diversity in the respiratory and intestinal tracts in individual patients in order to understand colonisation and transmission of MDROs, identify potentially protective components of the microbiome against MDROs	Characteristics of the microbiota will be determined by comparing alpha and beta diversity metrics between the groups of patients, alterations in the repertoire of antibiotic resistant genes, shifts in microbiota functional and metabolic capacity
	To study the impact of antibiotic utilisation on the ward-level multidrug resistant organism emergence and transmission dynamics	Prospective audit of antibiotic utilisation of participating wards, colonising bacteria in respiratory and stool samples, antibiograms of infective and colonising bacteria, whole genome and resistance

Mo Y, *et al. BMJ Open* 2021; 11:e050105. doi: 10.1136/bmjopen-2021-050105

		genes sequencing of multidrug resistant isolates, mathematical modelling using sequencing data to track the spread of pathogens between hosts allowing for unobserved infection times, multiple independent introductions of the pathogen, and within-host genetic diversity
	To construct predictive models with comprehensive clinical and molecular epidemiological data to produce setting-specific and evidence based antibiotic policies	Statistical models and patient screening algorithms in both low- to- middle and high income settings to predict outcomes including proportion of adequate empiric therapy, expected deaths, Quality Adjusted Life Years (QALY) loss, costs, and forecast trends in resistance

2. ABBREVIATIONS

CI	Chief Investigator
CRF	Case Report Form
ICU	Intensive care units
OxTREC	Oxford Tropical Research Ethics Committee
PI	Principal Investigator
VAP	Ventilator-Associated Pneumonia

Mo Y, *et al. BMJ Open* 2021; 11:e050105. doi: 10.1136/bmjopen-2021-050105

3. BACKGROUND AND RATIONALE

Hospitals and, in particular, intensive care units (ICUs) are epicenters for the emergence and dissemination of multidrug resistant (MDR) bacteria. ICUs have the most vulnerable patient population due to frequent use of invasive devices which break anatomical barriers, bypass host defences and distort normal protective microbiomes, because of high antimicrobial consumption. Consequently, the ICU population has one of the highest rates of nosocomial infections leading to a significant impact on healthcare costs, morbidity and survival [1]. With their controlled settings and well-defined patient populations, ICUs present an opportunity to conduct robust interventional and epidemiological studies on the effect of antimicrobial use on the emergence and spread of MDROs in individual patients as well as on the overall ecology in the ICU environment. Carefully conducted regional multi-centre trials in ICUs will provide important results that can potentially be extrapolated to larger clinical settings via epidemiological modelling to estimate the impact of the measures studied if these are well adopted for the whole Asian region.

VAP is the most common nosocomial infection in patients in ICUs. As demonstrated in a cross-sectional prevalence survey in 1,417 ICUs worldwide, the prevalence of respiratory tract infection was 64% among all patients with nosocomial infections [2]. Estimates of all- cause mortality in patients with VAP range from 20-50% [3,4], and can be as high as 94% in low- to middle-income countries [5]. VAP prolongs the length of mechanical ventilation by 7.6-11.5 days and prolongs hospitalisation by 11.5 - 13.1 days compared with similar patients without VAP, and the excess cost associated with VAP has been estimated at USD$40,000 per patient in the United States [6,7]. Given its high prevalence and associated antibiotic usage, VAP is likely to be a key driver of antimicrobial resistance (AMR) in ICUs.

Over the last few decades, we have continued to rely on the standard clinical, radiographic, and microbiological criteria with a low sensitivity and specificity of between 70 and 75% to diagnose VAP, respectively [8]. Identification of specific causative organisms is also difficult as the respiratory tract is non-sterile. Concordance between tracheal non-quantitative cultures and cultures of lung tissue from open lung biopsy was found to be as low as 40% [9]. These factors result in over-diagnosis and over-treatment of organisms thought to be causing VAP with empirical combinations of broad-spectrum antibiotics. For those patients who are on culture-directed definitive antibiotics, duration of treatment remains controversial. There are two notable clinical trials from France that have suggested that a short course of 8 days has comparable clinical efficacy as a long duration of 15 days [10,11]. However, these studies could not confidently conclude that the finding can be applied to VAP caused by non-fermenting Gram-negative bacilli due to increased recurrence in such patients. Important potential biases also exist in those studies. For example: long-course therapy is favoured due to the differential time period during which recurrence was assessed, persistent colonisation could have been erroneously classified as recurrent infections, and the observed non-significant high rate of recurrence in those caused by non-fermenting Gram-negative bacilli could be a spurious finding due to subgroup over-analyses [10,11]. This severely limits the applicability of the current recommendation in Asia. This is because the majority of VAP in this region are caused by non-fermenting Gram-negative bacilli such as *Pseudomonas aeruginosa* and *Acinetobacter baumannii* [12-15]. The current median number of days of antibiotic treatment remains at 12-13 days in Thailand [16, 17]. Currently available studies also suffer from narrowly defined inclusion criteria that excluded culture-negative VAP. This limits generalisability of the findings to VAP caused by unknown organisms. Therefore, it is crucial to define whether a short duration of antimicrobial therapy for VAP would provide equivalent clinical outcome and yield lower emergence of MDRO in Asian settings.

Because of the uncertainty about the exact causative organisms, ICUs are likely to utilise a high proportion of inappropriate empirical broad-spectrum antibiotics [17], which might contribute to the indirect 'collateral damage' of AMR emergence and dissemination to other patients. In Thailand, an alarming 95% of A. baumannii and 75% of P. aeruginosa isolates were recently found to be resistant to carbapenems, and 47% of K. pneumoniae isolates were ESBL-producing in VAP [18]. In Singapore, 91% of *A. baumannii* and 22% of all Gram-negative isolates collected during a 4-month period in 2010 were resistant to carbapenems in the "Comparative Activity of Carbapenem Testing" (COMPACT) study [19]. This emphasizes the importance of antimicrobial stewardship programmes (ASPs) which have shown to be capable of reducing broad-spectrum antibiotic consumption and potentially decreasing antibiotic resistance, particularly among Gram-negative pathogens [20-22]. However, current recommendations on ASP are based on very few conclusive studies as most ASP research was designed to evaluate cost-effectiveness instead of clinical outcomes such as reducing selection of MDR pathogens, mortality, hospital length of stay, and readmission rates [23-25]. In addition, operational delivery of ASPs is often hindered by cost, lack of diagnostics and data collection facilities, poor physician participation and lack of cooperative strategies [26]. Low uptake of ASPs, especially in SEA [27], calls for more rigorous and methodologically sound evaluations of ASP interventions to initiate practice and policy changes. Mathematical models offer tremendous potential for assessing the effectiveness of ASPs by removing the limitations inherent in human experimentation, including potential health risks, study cohort size and duration [28]. To date, the potential of modelling in evaluating ASPs in control of antimicrobial resistance is largely untapped and much work remains to be done to leverage this potential.

We aim to rationalise and reduce antibiotic consumption in ICUs and assess the benefit of this on both the individual patient and the overall cohort of ventilated patients. The specific aims are:

i) to conduct a randomised controlled trial to assess the clinical efficacy of a short duration of antibiotics (up to 7 days) versus prolonged antibiotic therapy (physician preference) in adults patients with VAP in Asia;

ii) to study the changes in individuals' microbiota and the impact on pathogen transmission dynamics and ward ecology with the use of various antibiotic regimens; and

iii) to characterise current antibiotic utilisation in the ICU and develop locally optimised, setting-specific empirical antibiotic policies.

This study includes adult patients (≥18 years old) who satisfy our inclusion criteria for VAP after ≥ 48 hours of mechanical ventilation.

Patient recruitment will be from:

1) National University Hospital, Singapore
2) Tan Tock Seng Hospital, Singapore
3) Sunpasitthiprasong Hospital, Ubon Ratchathani, Thailand
4) Srinagarind Hospital, Khon Kaen, Thailand
5) Patan Academy of Health Science, Patan Hospital, Kathmandu, Nepal

The results of the study will be generalizable to adult ventilated patients from both low-to-middle and high-income countries, especially those with VAP caused by Gram-negative bacteria.

4. OBJECTIVES AND OUTCOME MEASURES

Objectives	Outcome Measures	Timepoint(s) of evaluation of this outcome measure (if applicable)
**Primary Objective** To demonstrate clinical non-inferiority of a shorter antibiotic treatment duration compared to a longer duration in adult patients with VAP	Difference in the proportion of participants with the composite endpoint of death and VAP recurrence rate within 60(+5) days of enrolment	All outcome measures will be assessed within day 60(+5) of enrolment
**Secondary Objectives** To define other clinical benefits of shorter antibiotic treatment duration compared to a longer duration in adult patients with VAP	Ventilator-associated events, duration of mechanical ventilation, duration of hospitalisation, acquisition of multidrug resistant infection or colonisation during the hospitalisation, number of days of exposure to antibiotics during hospitalisation, number and types of extrapulmonary infections during hospitalisation (determined from cultures taken from sterile sites)	All outcome measures will be assessed within day 60(+5) of enrolment
To study the impact of various antibiotic regimen on the microbial community dynamics and diversity in the respiratory and intestinal tracts in individual patients in order to understand colonisation and transmission of MDROs, identify potentially protective components of the microbiome against MDROs	Characteristics of the microbiota will be determined by comparing alpha and beta diversity metrics between the groups of patients, alterations in the repertoire of antibiotic resistant genes, shifts in microbiota functional and metabolic capacity	Respiratory and stool samples will be collected from the study participants within 24 hours of enrolment, weekly during hospitalisation, and at day 28 (+5) and 60(+5) after enrolment
To study the impact of antibiotic utilisation on the ward-level multidrug resistant organism emergence and transmission dynamics	Prospective audit of antibiotic utilisation of participating wards, colonising bacteria in respiratory and stool samples, antibiograms of infective and colonising bacteria, whole genome and resistance	These will be measured throughout the duration of the

	genes sequencing of multidrug resistant isolates, mathematical modelling using sequencing data to track the spread of pathogens between hosts allowing for unobserved infection times, multiple independent introductions of the pathogen, and within-host genetic diversity	study in participating wards
To construct predictive models with comprehensive clinical and molecular epidemiological data to produce setting-specific and evidence based antibiotic policies	Statistical models and patient screening algorithms in both low-to- middle and high income settings to predict outcomes including proportion of adequate empiric therapy, expected deaths, Quality Adjusted Life Years (QALY) loss, costs, and forecast trends in resistance	N.A.

5. STUDY DESIGN

The study design is a randomised, partially double-blinded (blinding may be achieved up to 7 days) controlled non-inferiority trial to assess the efficacy of short vs. longer antibiotics course in adults with VAP in Asia.

Screening and inclusion criteria

We will apply the US Centers for Disease Control and Prevention (CDC) National Healthcare Safety Network (NHSN) VAP diagnostic criteria on patients who have been mechanically ventilated for ≥48 hours as our study subject inclusion criteria [29]. While we acknowledge that there is no "gold standard" diagnostic criteria to VAP and that clinical criteria correlate poorly with autopsy findings (previously determined to be 69 % sensitivity and 75 % specificity [30]), the CDC NHSN diagnostic criteria is sensitive and practical for use in ICUs of various resources and settings. Only one episode of suspected VAP will be included for this study.

We agree with the 2016 Clinical Practice Guidelines by the Infectious Diseases Society of America (IDSA) and the American Thoracic Society (ATS) guideline that the additional use of infection scores and other biomarkers including procalcitonin, c-reactive protein, and soluble triggering receptor expressed on myeloid cells-1 lack sensitivity and specificity and will not be included in our inclusion criteria [31].

Microbiological culture results are not part of our inclusion criteria so that we are able to recruit patients who have suspected VAP but respiratory cultures are negative. Management strategies for this group of patients have not been defined by a randomised study. There are two observational studies that compared outcomes among those whose antibiotics were withheld on the basis of negative respiratory cultures to those whose antibiotics were continued [32, 33]. Patients whose antibiotics were discontinued did not have a higher mortality or rate of new respiratory infection compared to patients whose antibiotics were continued.

Mo Y, et al. BMJ Open 2021; 11:e050105. doi: 10.1136/bmjopen-2021-050105

Randomisation and Intervention

Following screening and fulfilling the inclusion/exclusion criteria, the study subject will be recruited within 72 hours. Antibiotic treatment course will be determined according to randomisation.

Participants in the intervention (short duration) arm will receive antibiotics for up to 7 days. Antibiotics should be stopped from day 3 to 7 if respiratory cultures are negative and the patients fulfil a set of stringent clinical criteria signifying cardiopulmonary stability for 48 hours. If the respiratory cultures are positive, patients who fulfil the same set of clinical criteria should have their antibiotics stopped from day 5 to 7 (see section 8. Interventions). Antibiotics administered via all routes i.e. intravenous, oral and nebulisation should be stopped. Randomisation will be carried out when the participants fulfil the clinical stop criteria. The rationale for stopping antibiotics at the above time points is as follows:

Group 1: Short Duration arm

(a) Day 3
Patients who have negative respiratory cultures should have their antibiotics stopped from day 3 to 7 if the patient satisfies a set of criteria signifying clinical stability. As discussed above, though there are no randomised studies to support this, there are 2 observational studies [32, 33] showing that discontinuation of antibiotics in these cases did not result in a higher mortality or rate of new respiratory infections compared to those whose antibiotics were continued. In addition, those whose antibiotics were withheld had a lower rate of total secondary infections by MDROs. One of these studies concluded that antibiotics could be safely discontinued in patients with negative respiratory cultures with no significant impact on mortality, despite persistence of signs and symptoms of pneumonia in 35% of these patients [33]. The above studies have led to the recommendation in the 2016 IDSA/ATS guideline that for patients with suspected VAP whose respiratory invasive culture results are below the diagnostic threshold for VAP, antibiotics should be withheld rather than continued [31].

(b) Day 5-7
The 2016 IDSA/ATS VAP guideline recommends a 7-day course of antimicrobial therapy for the treatment of VAP[31]. However, 7-day course is empirical and durations shorter than 7 days have been studied in randomised trials. One of these studies by Singh et al [34] evaluated 3 days of empirical ciprofloxacin monotherapy for patients who satisfy a set of clinical criteria signifying low likelihood of active VAP at day 3 of treatment. Compared to those who received longer duration of antibiotics, there was no difference in mortality or ICU length of stay. Patients in the short duration group had less antibiotic resistance and fewer superinfections. Another randomised study by Micek et al [35], adopted an antibiotic discontinuation policy to shorten VAP treatment. Similarly there was no difference between the short (6.0 ± 4.9 days) and long duration treatment groups in terms of mortality and VAP recurrences. The majority of the causative pathogens in this study are Gram-negative bacteria including Pseudomonas and Acinetobacter, which are also the commonest VAP pathogens in Asia [35]. The above evidence supports the use of a set of clinical stability criteria to establish the duration of antibiotic treatment for VAP.

The set of clinical stability criteria used in this study is adopted from Micek et al [35], and IDSA/ATS consensus guidelines on the management of community-acquired pneumonia in adults [36]. The

community-acquired pneumonia guideline is used because a similar set of clinical stability criteria was not defined in the IDSA/ATS VAP guideline.

Group 2: Long Duration arm

Participants in the control (long duration) arm will receive standard care, which is antibiotic treatment for at least 8 days with the exact duration decided by the primary physician.

Sample and data collection

Respiratory samples (at least 1 set) will be collected from the participants within 24 hours of enrolment, if not yet collected by the managing physician, for microbiological cultures. Respiratory cultures will be collected either via the endotracheal tube (ETT) or bronchoalveolar lavage (BAL) as ordered by the primary physicians. Both non-invasive (ETT) and invasive (BAL) samples are acceptable methods for microbiological diagnosis of VAP. No difference in mortality in the invasive versus non-invasive groups was shown in large randomised controlled studies including the Canadian Clinical Trials study [37], and subsequently a Cochrane meta-analysis [38] of 1,367 patients with VAP. Bacterial isolates grown from all other routine clinical cultures such as blood, cerebral spinal fluid, pus etc, will be retrieved from the microbiology laboratory and frozen and stored. These isolates will undergo whole genome sequencing to compare the within-host evolution of resistant organisms.

For microbiome studies, 1 set of respiratory and stool samples will be collected within 24 hours and weekly during hospitalisation for DNA extraction and microbiome analysis.

We will collect relevant clinical and microbiological data from the participants throughout their hospitalisation. Data collection will be done via review of medical records, microbiological culture results and drug administrative charts.

Concurrently, we will collect prospective data on antibiotics utilisation, adaption and compliance of VAP prevention bundles and hand hygiene rates in the participating units. These audits will include patients who are not study participants. These data are important as they are potential confounders to our outcome measures including mortality, VAP recurrence rate, duration of mechanical ventilation, acquisition of MDRO infection, and number and types of extrapulmonary infections during hospitalisation. Consents will not be required for these patients not directly participating in the study because these audits are standard procedures in hospitals.

Follow-up visits

The composite endpoint of death and VAP recurrence rate is assessed at 60(±5) days of enrolment done via an inpatient or outpatient follow-up visit. The time point of day 60(±5) is chosen, in contrast with day 28 in previous studies, to avoid a bias in favouring long duration of treatment in assessment of VAP recurrences. A longer time point is deemed impractical and may contribute to high loss-to-follow-up rates especially for patients who reside in rural and remote areas.

Two follow-up visits are scheduled at day 28(±5) and 60(±5). One set of respiratory and stool sample will be collected from all participants. The expected duration of participant participation is 60(±5) days from recruitment. Please see appendix B for schedule of study procedures.

6. PARTICIPANT IDENTIFICATION AND RECRUITMENT

6.1. Study Participants

Adult patients (≥18 years old) in participating wards who have been on mechanical ventilation for more than 48 hours.

6.2. Inclusion Criteria

a. Patients 18 years and older
b. Invasive mechanical ventilation ≥ 48 hours
c. Satisfy the US Centers for Disease Control and Prevention National Healthcare Safety Network VAP diagnostic criteria [29]
  - At least one of the following:
    1. temperature > 38 °C
    2. white blood cell count ≥ 12,000 cells/mm^3 or ≤ 4,000 cells/mm^3
    3. altered mental status with no other causes in >70 year-olds; AND
  - Two or more chest imaging tests demonstrating at least one of the following:
    1. new and progressive OR progressive and persistent infiltrate
    2. new and persistent OR progressive and persistent consolidation
    3. new and persistent OR progressive and persistent cavitation, AND
  - At least two of the following:
    1. new onset of purulent sputum, or change in character of sputum, or increased respiratory secretions, or increased in suctioning requirements
    2. new onset or worsening tachypnea or dyspnea
    3. rales or bronchial breath sounds
    4. worsening gas exchange defined by oxygen desaturations (e.g., PaO2/FiO2 <240), increased oxygen requirements or increased ventilation demand

6.3. Exclusion Criteria

a. Poor likelihood of survival as defined by a Sepsis-related Organ Failure Assessment score (SOFA score) of >11 points [39]
b. Immunocompromised patients (HIV with CD4 <200 cells/mm^3, corticosteroids> 0.5 mg/kg per day for > 30 days, received chemotherapy in the past 3 months, solid organ or hematopoietic cell transplant)
c. Patients receiving antibiotic therapy for any other defined extra-pulmonary infections that warrant a duration of antibiotics longer than 7 days or complications of pneumonia such as lung abscess or empyema that warrant a duration of antibiotics longer than 7 days (excluding anti-tuberculosis treatment, antifungal medications, antibiotics meant for chronic suppression of chronic infections or chronic obstructive lung disease)
d. Patients who have been treated for VAP for more than 7 days from screening
e. Vulnerable population including prisoners and refugees

7. STUDY PROCEDURES

7.1. Recruitment

Relevant sub-specialists such as general medicine, respiratory, infectious disease, intensive care and anaesthesia physicians, nurses and pharmacists involved in the care of ventilated patients from all

participating sites will be briefed on the study protocol in advance. With their support, a site PI will be nominated to head the study.

The study team will do screening for potential subjects. Potential subjects are identified according to the eligibility criteria stated above. When a potential subject is identified, our study team will approach the primary physician for his/ her permission for the study team to speak to the patient or patient's legal representative or patient's next-of-kin (NOK) for consent. Patient's legal representative or NOK may be then contacted via phone call or in person. Discussion and explanation of the study procedures and signing of the consent will be done face-to-face.

7.2. Screening and Eligibility Assessment

Screening will be done on all mechanically ventilated patients in the respective study sites according to the eligibility criteria stated above. Participants should be recruited into the study as soon as possible after screening. A maximum of 72 hours is allowed between screening (fulfilment of the inclusion/exclusion criteria) and recruitment.

7.3. Informed Consent

Participants' legal representatives or NOKs will be asked to represent the participants if the participants are sedated and do not have the capacity to make informed decision to participate in the study. The participants, their legal representatives or NOKs must personally sign and date the latest approved version of the Informed Consent form before any study specific procedures are performed.

Written and verbal versions of the Participant Information and Informed Consent will be presented to the participants, their legal representatives or NOKs detailing no less than: the exact nature of the study; what it will involve for the participant; the implications and constraints of the protocol; the known side effects and any risks involved in taking part. It will be clearly stated that the participant, his/her legal representative or NOK (on behalf of the participant) is free to withdraw from the study at any time for any reason without prejudice to future care, and with no obligation to give the reason for withdrawal.

The participants, his/her legal representatives or NOKs will be allowed sufficient time to consider the information, and the opportunity to question the Investigator or other independent parties to decide whether they will participate in the study. Written Informed Consent will then be obtained by means of participants', their legal representatives' or NOKs' dated signatures and dated signature of the person who presented and obtained the Informed Consent. The person who obtained the consent must be suitably qualified and experienced, and have been authorised to do so by the Investigator. A copy of the signed Informed Consent will be given to the participants, their legal representatives or NOKs. The original signed form will be retained at the study site.

When the participants are deemed to have decision-making capacity by the primary physicians, he/she will be re-consented with the same procedures as above.

The concurrent prospective data on clinical cultures, antibiotics utilisation, adaption and compliance of VAP prevention bundles and hand hygiene rates will involve all participating wards throughout the period of the study. All patients admitted to these units will be included as part of this audit and consents will not

be taken for this purpose. All information collected as part of the audit will be anonymised to ensure patient confidentiality.

7.4. Randomisation, blinding and unblinding

Randomisation will be done via stratified block randomisation by the study sites to ensure participants with similar characteristics such as gender and age are distributed equally in the intervention and control groups. Randomisation will be done with a computer programme with a seed to allow reproducibility. Randomisation will be done with a 1:1 ratio. To prevent predictability of the random sequence, generation of the randomisation sequence is performed by an independent statistician and details of the randomisation generation is unavailable to all investigators. Randomisation will be allocated using sequentially numbered opaque envelopes. Fitness criteria for randomisation (section 8) must be met prior to randomisation.

Patients will be blinded to the study, as they will not be informed of the treatment duration and likely to be sedated and unaware of the treatment regimens. Investigators will be blinded during the assessment of the participants for clinical stability based on the above-described criteria to minimise observer bias. Once conditions for stopping antibiotics are satisfied, the investigator will be unblinded and contact the primary physicians to stop antibiotics. The physicians will remain blinded until they are informed that the participant is suitable to stop antibiotics. Independent assessors, who are assigned to determine pneumonia recurrences, will be blinded from the randomisation arms. This will be achieved by blinding all study details, including randomisation arms, for participants with potential recurrences from these independent assessors.

7.5. Baseline Assessments

At recruitment, participants' demographics, medical history, antibiotics administration record, chest X-ray or other imaging findings, biochemical, microbiological and haematological results and clinical parameters will be collected. These are routine investigations ordered by the clinical teams taking care of the patients. Stool samples and upper and lower respiratory tract samples will be collected if they have not already been collected in the 24 hours prior to recruitment.

7.6. Subsequent Visits

Clinical information will be collected during participant's hospitalisation. Two follow-ups will be done on day 28(±5) and 60(±5) after recruitment. During these visits, similar clinical information will be collected (if available) via hospital record review. Repeat respiratory and stool sample will also be collected. In the event that the participants are not able to attend the follow-ups in person, a telephone interview can be done in place of a physical visit.

7.7. Sample Handling

Respiratory cultures will be collected with the usual volume of 5-10ml per sample. Respiratory cultures may be collected via ETT, BAL or any other methods that the primary physician and local microbiology laboratories routinely use. Stool culture will be collected following defecation with the usual volume of 10-

50g per sample. Stool samples are collected at recruitment, weekly during hospitalisation and day 28/60(±5) after enrolment.

Microbiology cultures will be processed and reported in the local laboratories, which are expected to have standing quality control measures. Isolates, once identified, will be preserved, frozen and transported to an Oxford or Singapore study laboratory for genomic studies. These genomic results should concur with the initial microbiology laboratories' identification of the isolates.

a) Respiratory cultures
All samples will be cultured and identified via standard microbiological methods. Susceptibility studies will be performed using EUCAST or CLSI agar method and breakpoints. Culture results with antibiogram are usually available within 48 to 72 hours after sample collection.

Pathogens causing VAP and colonising MDROs will be processed for DNA extraction and undergo whole genome sequencing. These sequencing data will be used to track the spread of pathogens between hosts allowing for unobserved infection times, multiple independent introductions of the pathogen, and within-host genetic diversity.

These isolates will be de-identified and stored for up to 15 years for use in future ethically approved studies. We will seek permission from the participants to store their samples and include this in the informed consents.

b) Respiratory and stool samples
For microbiome analysis, respiratory and stool samples will be processed for DNA extraction. Shotgun metagenomics approach will be used to characterise microbial community dynamics in the respiratory and intestinal tract. The characteristics of the microbiota will be determined by comparing alpha and beta diversity metrics between the groups of patients to explore the short- and long- term impact of the various durations of antibiotics in individual patients. The data will also be used to assess if alterations in the repertoire of antibiotic resistant genes can be detected, and to study shifts in functional and metabolic capacity during the time course with a view to getting mechanistic insights into potentially protective components of the microbiome.

These isolates will be de-identified and stored for up to 15 years for use in future ethically approved studies. We will seek permission from the participants to store their samples and include this in the informed consents.

c) Bacterial isolates from other routine clinical cultures such as blood, cerebral spinal fluid, pus etc

All bacterial isolates including MDR *Acinetobacter spp, Pseudomonas spp, Enterobacteriaceae, Staphylococcus aureus and Enterococcus spp*, grown from other routine clinical cultures will be de-identified and stored for up to 15 years for use in future ethically approved studies. In the current study, these isolates will undergo whole genome sequencing to compare the within-host evolution of resistant organisms. We will seek permission from the participants to store their samples for up to 15 years in the informed consents.

7.8. Discontinuation/Withdrawal of Participants from Study

Each participant or NOK has the right to withdraw from the study at any time. The reason for withdrawal will be recorded in the Case Report Form. Withdrawal from the study will not result in exclusion of the data for that participant from analysis.

In addition, the Investigator may discontinue a participant from the study at any time if the Investigator considers it necessary for any reason including but not limited to:

- Ineligibility (either arising during the study or retrospectively having been overlooked at screening)
- Significant protocol deviation
- Significant non-compliance with treatment regimen or study requirements
- Loss to follow up
- Failure to meet fitness criteria by day 7

We will compare baseline characteristics between patients who had protocol violations or who were unavailable for follow-up with those who did not. Follow-ups and data collection listed on the standard CRF for discontinued participants, including those who fail to be randomised, will be carried out within day 60(±5) of enrolment.

Discontinued subject can be rescreened (under a new number). If the subject is rescreened, a new subject number will be allocated. The above patients will not be followed up once withdrawn or discontinued, and replaced to achieve the calculated sample size.

7.9. Definition of End of Study

The end of study is the date of the last follow-up visit of the last participant.

8. INTERVENTIONS / INVESTIGATIONS

Antibiotic treatment for VAP will be tailored to the susceptibility of the pathogen(s) and in accordance to the 2016 IDSA/ATS VAP guideline [31]. Primary physicians are encouraged to convert initial empirical regimen to narrow- spectrum therapy based on culture results. In culture-negative cases, empirical antibiotic choice should be made depending on local antibiogram. The study team will not intervene on the antibiotics choices as this is beyond the objectives of the study.

Number of days of antibiotics is calculated from the first day of appropriate coverage according to the susceptibility of at least 1 of the pathogen(s) recovered from respiratory cultures taken within 48h of screening or VAP symptom onset.

For those participants randomised to the intervention (short duration) arm, site investigators should assess the patients daily. Antibiotics should be stopped at

a) day 3 to 7, if all respiratory cultures during the same episode of VAP are negative, given that the criteria below are satisfied,

b) day 5 to 7, if any of the respiratory cultures are positive is positive attributable to the current episode of VAP, given that the fitness criteria below are satisfied,

a. body temperature was ≤ 38.3°C (core body temperature measured orally or rectally) or 38.0°C (axillary) for 48 hours, and

b. hemodynamic instability (systolic blood pressure ≥ 90 mm Hg without inotropic support or no requirement of inotropic support to maintain systolic blood pressure above 90 mm Hg).

In the short duration arm, all antibiotics should be withdrawn by day 7 (short duration) according to the randomisation assignment, except those participants with treatment failure in the case of persistent VAP or a new-onset infection of a different source prior to the last day of assigned duration of antibiotics. Persistent VAP or treatment failure is defined as the lack of improvement of hemodynamic stability or ventilation requirements without an alternative cause other than the same episode of VAP. These patients will be continued to be monitored for the above-described "fitness criteria" and antibiotics should be stopped when they eventually meet the criteria. Recurrent VAP is defined as an additional episode of VAP satisfying the CDC clinical and radiological criteria of VAP [29] following commencement of the primary VAP treatment within 60(±5) days of enrolment. Opinion from 2 respiratory experts blinded to the randomisation will be sought to diagnose persistence and recurrences. Patients who are diagnosed with an alternative source of infection or a complication from VAP such as lung abscess or empyema that warrant a duration of antibiotics longer than 7 days prior to the last day of assigned duration of antibiotics are not considered treatment failures. These patients will not meet fitness criteria for randomisation and be discontinued from the study and subsequent analysis (see section 7.8). In cases where patients randomised to short duration and satisfy the above-described "stop criteria" but did not have their antibiotics stopped by day 7, they are considered protocol deviations. They will be analysed with intention to treat analysis.

Duration of antibiotics in control group will be at least 8 days with the exact duration decided by the managing physicians.

Adverse events will be recorded throughout the study period, including C. difficile infection, acute kidney injury, hepatitis, drug allergies, haematological and other complications.

9. SAFETY REPORTING

Participants in the shorter treatment duration arm may experience an increase in incidence of VAP relapse. Participating in the study will not delay the participants from receiving antibiotic treatment for recurrent VAPs. Decision to treat these participants for VAP recurrence will be made by the primary physicians.

For outcome measurement purposes, opinion from 2 respiratory experts blinded to the randomisation will be sought to diagnose recurrences according to the above-described clinical and radiological criteria of VAP. Interim analysis will be done to ensure that participants in the shorter treatment duration arm are not experiencing a significant increase in incidence of ventilator-associated pneumonia relapse.

9.1. Definition of Serious Adverse Events

A serious adverse event is any untoward medical occurrence that:

- results in death
- is life-threatening
- requires inpatient hospitalisation or prolongation of existing hospitalisation
- results in persistent or significant disability/incapacity

Other 'important medical events' may also be considered serious if they jeopardise the participant or require an intervention to prevent one of the above consequences.

NOTE: The term "life-threatening" in the definition of "serious" refers to an event in which the participant was at risk of death at the time of the event; it does not refer to an event which hypothetically might have caused death if it were more severe.

9.2. Reporting Procedures for Serious Adverse Events

Given that VAP in ICU cohorts is associated with high mortality of 13.6–42.8% [42] and high recurrence rate previously shown in Southeast Asia, we would expect 55%-60% of our estimated sample size of 460 patients to fulfil the above definition of SAE. This constitutes up to 276 (138 in each arm) participants that would require SAE reporting.

As these events are very frequent and expected outcomes from ICU cohorts of patients, local Investigators will report mortalities and VAP recurrences that were 'definitely related' (definitely resulted from shortening antibiotic therapy duration), 'probably related' (some evidence that the event was resulted from shortening antibiotic therapy duration), 'possibly related' (little evidence that the event was resulted from shortening antibiotic therapy duration), and 'not related' (no evidence that the event was resulted from shortening antibiotic therapy duration) to CI on a monthly basis in the form of collated SAE reports. CI will, in turn, present these collated SAE reports to a nominated member of Data and Safety Monitoring Committee (DSMC) on a monthly basis.

Local investigator will present these SAE to the relevant local authorities according to local reporting requirements and timelines.

To address the main risk of our study, which is that the participants in the intervention arm (short duration) may have higher recurrence and mortality rates, we have planned for four interim analysis for safety monitoring by the Data and Safety Monitoring Committee (DSMC).

10. STATISTICS AND ANALYSIS

10.1. Description of Statistical Methods for Primary and Secondary Outcomes

Descriptive statistics will include frequency tables for categorical data, means (SDs), or medians (interquartile ranges) depending on the distribution of the data. Categorical variables will be compared with $\chi 2$ and Fisher exact tests as appropriate and continuous variables with unpaired, 2-tailed t tests or nonparametric Wilcoxon rank sum tests as appropriate.

The primary and secondary outcomes of the study populations will be analyzed using both unadjusted and adjusted methods in both the per-protocol and intention-to-treat populations. Adjustment will be done with inverse probability weighting, using baseline patient characteristics (study site, age, gender, comorbidities, residence prior to admission, type of ICU admitted to, SOFA score, VAP infection with CRE, maximum heart rate and minimum mean arterial blood pressure on randomisation day, duration of intubation prior to developing VAP, reason for intubation) as independent variables. Non-inferiority will be concluded if the upper limit of the one-sided 95% confidence intervals from both adjusted analyses do not cross the non-inferiority margin. The purpose of using both adjusted analyses on the intention-to-treat and per-protocol populations to determine non-inferiority is to minimise the inflation of type 1 error associated with non-adherence in non-inferiority trials. [40]

10.2. Definitions of study populations

Intention-to-treat: The intention-to-treat population includes all study participants who have been randomised during the conduct of the study

Per-protocol: The per-protocol population includes all study participants who fulfill eligibility criteria specified in the inclusion/exclusion criteria (section 6.2 and 6.3), fitness criteria for randomisation (section 8) and received 7 days or less of appropriate antibiotics in the short arm, and 8 days or more of appropriate antibiotics in the long arm.

10.3. Interim analyses

Four interim analyses will be performed on the primary endpoint whenever 25% of patients has been randomised and have completed the 60 (±5) days follow-up. The interim analyses will be performed by a trial statistician in coordination with the study team. The statistician will report to the independent data and Data and Safety Monitoring Committee (DSMC). The DSMC will have unblinded access to all data and will discuss the results of the interim-analysis during a DSMC meeting. A steering committee will also be constituted and will decide on the continuation of the trial and will report to the central ethics committee. We will use the group sequential design adopting the boundaries proposed by Fleming- Harrington- O'Brien (R=0.8) [41] to terminate the trial prematurely once the Z value exceeds the defined boundaries.

10.2. The Number of Participants

The study is designed to demonstrate the non-inferiority on the composite endpoint of mortality and recurrence at 60(±5) days of the short duration versus the long duration of antibiotic treatment for VAP. A meta-analysis showed that mortality attributable to VAP ranges from 13.6–42.8% in Southeast Asia [42]. Considering that our primary outcome is a composite binary outcome of mortality and recurrence of VAP, we estimate this to be 55%. We derived an absolute non-inferiority margin of 12% with the fixed-margin method, preserving at least 50% of the efficacy of standard treatment in VAP. Using a group sequential design adopting the boundaries proposed by Fleming- Harrington- O'Brien (R=0.8) [41], a maximum of 412 patients will be required to achieve a power of 80% to conclude non-inferiority between the two groups with an one-sided α risk of 5%. As we anticipate a loss to follow-up of up to 10%, we plan to enrol a maximum of 460 patients.

10.3. Analysis of microbiota and whole genome sequencing

Patients will be stratified into groups according to types and duration of antibiotic exposure. Shotgun metagenomics approach can be used to characterise microbial community dynamics in the respiratory and intestinal tract. The characteristics of the microbiota will be determined by comparing alpha and beta diversity metrics between the groups of patients and healthy volunteers to explore the short- and long-term impact of the various durations of antibiotics in individual patients. The data will also be used to assess if alterations in the repertoire of antibiotic resistant genes can be detected, and to study shifts in functional and metabolic capacity during the time course with a view to getting mechanistic insights into potentially protective components of the microbiome.

11. DATA MANAGEMENT

11.1. Access to Data

Direct access will be granted to authorised representatives from the University of Oxford and any host institution for monitoring and/or audit of the study to ensure compliance with regulations.

11.2. Data Handling and Record Keeping

All study data will be entered on MACRO, the Clinical Data Management System. The study database will be developed according to the approved Case Report Forms (CRFs). As for documents that contain patient identifiers, only the site PIs will have access to these documents.

The MORU Data Management Standard Operating Procedures will be followed in the study. After the study is closed, the study documents will be kept in the accessible storage on request for 15 years. The final dataset will be archived in the data repository after the publication.

12. QUALITY CONTROL AND QUALITY ASSURANCE PROCEDURES

The study will be conducted in accordance with relevant regulations and standard operating procedures.

12.1 Study monitoring

An appointed study monitor from the MORU clinical trial support group and a project coordinator will regularly visit the study sites for quality control. All study sites will be assessed prior to initiation of the study for capacity to conduct the randomised controlled trial, during the study and upon completion to ensure data quality. After site initiation, monitoring visits will perform monitoring on informed consent forms, CRF for completeness and accuracy of data and sample storage. There will be a minimum of three monitoring visits per study site: after 3-5 participants enrolled, after 50% of the target sample size enrolled for a particular site and upon study completion. Monitoring reports will be made available to the study sites and investigators after each visit.

12.2 Strategies to improve adherence

Regular meetings with the study sites will be carried out to ensure buy-in from the physicians and healthcare providers. Their feedback will be sought throughout the study, and improvements will be made

in the study procedures to maintain ongoing support from the local ICUs. The study team will contact the primary physicians prior to enrolment and randomisation to ensure their adherence to allocated interventions.

13. ETHICAL AND REGULATORY CONSIDERATIONS

13.1. Declaration of Helsinki

The CI will ensure that this study is conducted in accordance with the principles of the Declaration of Helsinki.

13.2. Guidelines for Good Clinical Practice

The CI will ensure that this study is conducted in accordance with relevant regulations and with Good Clinical Practice.

13.3. Approvals

The protocol, participant information sheet and informed consent form will be submitted to OxTREC and local ethics committees for written approval.

The Investigator will submit and, where necessary, obtain approval from the above parties for all amendments to the original approved documents.

13.4. Participant Confidentiality

The study staff will ensure that the participants' anonymity is maintained. The participants will be identified only by a participant ID number on all study documents and any electronic database, with the exception of the CRF, where participant initials will be added. All documents will be stored securely and only accessible by study staff and authorised personnel. The study will comply with the Data Protection Act, which requires data to be anonymised as soon as it is practical to do so.

13.5. Expenses and Benefits

The study team will reimburse reasonable travel expenses for any visits additional to normal care.

13.6. Reporting

The CI shall submit an Annual Progress Report to OxTREC and local ethics committees on the anniversary of the date of approval of the study. In addition, the CI shall submit an End of Study Report to OxTREC and local ethics committees.

14. FINANCE AND INSURANCE

14.1. Funding

The project is jointly funded by Medical Research Council/ Department for International Development (MRC/DfID) (Grant Ref: MR/K006924/1), and Singapore National Medical Research Council (Grant ref: CTGIIT18may-0003).

14.2. Insurance

Specialist insurance policy is in place, which would operate in the event of any participant suffering harm as a result of their involvement in the research (Newline Underwriting Management Ltd, at Lloyd's of London).

15. PUBLICATION POLICY

The CI will coordinate writing and reviewing of drafts of the manuscripts, abstracts, and any other publications arising from the study. Authorship will be determined in accordance with the ICMJE guidelines and other contributors will be acknowledged.

16. REFERENCES

1. Brusselaers N, Vogelaers D, Blot S. The rising problem of antimicrobial resistance in the intensive care unit. *Annals of Intensive Care* 2011;1:47. doi:10.1186/2110-5820-1-47.
2. Vincent JL, Rello J, Marshall J, et al. International study of the prevalence and outcomes of infection in intensive care units. *JAMA* 2009;302:2323-2329.
3. Kalil AC, Metersky ML, Klompas M, et al. Management of Adults With Hospital-acquired and Ventilator-associated Pneumonia: 2016 Clinical Practice Guidelines by the Infectious Diseases Society of America and the American Thoracic Society. *Clin Infect Dis* 2016;63(5):e61.
4. Stebbings AE, Ti TY, Tan WC. Hospital acquired pneumonia in the medical intensive care unit--a prospective study. *Singapore Med J*. 1999 Aug;40(8):508-12.
5. Y Arabia, N Al-Shirawia, Z Memishb, et al. Ventilator-associated pneumonia in adults in developing countries: a systematic review *Int J Infec Dis* 2008;12(5): 505–512.
6. Muscedere JG, Day A, Heyland DK. Mortality, attributable mortality, and clinical events as end points for clinical trials of ventilator-associated pneumonia and hospital-acquired pneumonia. *Clin Infect Dis* 2010;51:S120-5.
7. Kollef MH, Hamilton CW, Ernst FR. Economic impact of ventilator-associated pneumonia in a large matched cohort. *Infect Control Hosp Epidemiol* 2012;33(3):250-6.
8. N Fabregas, S Ewig, A Torres, et al. Clinical diagnosis of ventilator associated pneumonia revisited: comparative validation using immediate post-mortem lung biopsies. *Thorax* 1999; 54:867-873.
9. Hill JD, JL Ratliff, J C Parrott, et al. Pulmonary pathology in acute respiratory insufficiency: lung biopsy as a diagnostic tool. *J Thoracic Cardiovas Surg* 1976;71:64-71.
10. Chastre J, Wolff M, Fagon JY, et al. Comparison of 8 vs 15 days of antibiotic therapy for ventilator-associated pneumonia in adults: a randomized trial. *JAMA* 2003; 19;290(19):2588-98.
11. Capellier G, Mockly H, Charpentier C, et al. Early-Onset Ventilator-Associated Pneumonia in Adults Randomized Clinical Trial: Comparison of 8 versus 15 Days of Antibiotic Treatment. *PLoS ONE* 2012; 7(8): e41290.
12. C Luyt, N Bréchot, J Trouillet et al. Antibiotic stewardship in the intensive care unit. *Crit Care* 2014;18:480.
13. Jones RN. Microbial etiologies of hospital-acquired bacterial pneumonia and ventilator-associated bacterial pneumonia. *Clin Infect Dis* 2010;51 Suppl 1:S81-7.
14. Nhu NT, Lan NP, Campbell JI, et al. Emergence of carbapenem-resistant *Acinetobacter baumannii* as the major cause of ventilator-associated pneumonia in intensive care unit patients at an infectious disease hospital in southern Vietnam. *J Med Microbiol.* 2014;63:1386–1394.
15. A Vasudevan, L Chuang, J Li, A Mukhopadhyay, EYY Goh, PA. Tambyah. Inappropriate empirical antimicrobial therapy for multidrug-resistant organisms in critically ill patients with pneumonia is not an independent risk factor for mortality: Results of a prospective observational study of 758 patients. *J Glob Antimicrob Resist.* 2013;3: 123–130.
16. P Rattanaumpawan, J Lorsutthitham, P Ungprasert, N Angkasekwinai, V Thamlikitkul. Randomized controlled trial of nebulized colistimethate sodium as adjunctive therapy of ventilator-associated pneumonia caused by Gram-negative bacteria. *J Antimicrob Chemother*. 2010; 65(12): 2645-2649.
17. Inchai J, Pothirat C, Liwsrisakun C, Deesomchok A, Kositsakulchai W, Chalermpanchai N. Ventilator-associated pneumonia: epidemiology and prognostic indicators of 30-day mortality. *Jpn J Infect Dis.* 2015;68(3):181-6.

18. W Reechaipichitkul, S Phondongnok, J Bourpoern, et al. Causative agents and resistance among hospital-acquired and ventilator-acquired pneumonia at Srinagarind Hospital, Northeastern Thailand. *Southeast Asian J Trop Med Public Health* 2013;44(3):490-502.
19. Kiratisin, Pattarachai *et al*. Comparative in vitro activity of carbapenems against major Gram-negative pathogens: results of Asia-Pacific surveillance from the COMPACT II study. *Int J Antimicrob Agents*. 2012;39(4):311-6.
20. White AC Jr, Atmar RL, Wilson J, Cate TR, Stager CE, Greenberg SB. Effects of requiring prior authorization for selected antimicrobials: expenditures, susceptibilities, and clinical outcomes. *Clin Infect Dis.* 1997;25(2):230-9.
21. Pakyz AL, Oinonen M, Polk RE. Relationship of carbapenem restriction in 22 university teaching hospitals to carbapenem use and carbapenem-resistant Pseudomonas aeruginosa. *Antimicrob Agents Chemother* 2009; 53:1983–6.
22. Lewis GJ, Fang X, Gooch M, Cook PP. Decreased resistance of Pseudomonas aeruginosa with restriction of ciprofloxacin in a large teaching hospital's intensive care and intermediate care units. Infect Control Hosp Epidemiol 2012; 33:368–73.
23. Jessina C. McGregor, J P Furuno. Antimicrobial stewardship: Patients over process: Optimizing Research Methods Used for the Evaluation of Antimicrobial Stewardship Programs. *Clin Infect Dis.* 2014;59(S3): S185-S192.
24. Schuts EC, Hulscher ME, Mouton JW *et al*. Current evidence on hospital antimicrobial stewardship objectives: a systematic review and meta-analysis. *Lancet Infect Dis.* 2016;16(7):847-56.
25. Kardaś-Słoma L, Boëlle P-Y, Opatowski L, Guillemot D, Temime L. Antibiotic Reduction Campaigns Do Not Necessarily Decrease Bacterial Resistance: the Example of Methicillin-Resistant Staphylococcus aureus. *Antimicrob Agents Chemother*. 2013;57(9):4410-4416.
26. Bal AM, Gould IM. Antibiotic stewardship: overcoming implementation barriers. *Curr Opin Infect Dis.* 2011;24(4):357-62.
27. Howard P, Pulcini C, Levy Hara G, *et al.* An international cross-sectional survey of antimicrobial stewardship programmes in hospitals. *J Antimicrob Chemother* 2015; 70: 1245–55.
28. L Caudill, JR Wares. The Role of Mathematical Modeling in Designing and Evaluating Antimicrobial Stewardship Programs. *Curr Treat Options Infect Dis.* 2016; 8:124–138.
29. Centers for Disease Control and Prevention. Pneumonia (Ventilator-associated [VAP] and non-ventilator-associated Pneumonia [PNEU]) Event. January 2017. Retrieved from https://www.cdc.gov/nhsn/pdfs/pscmanual/6pscvapcurrent.pdf
30. Fabregas N, Ewig S, Torres A, Al-Abiary M, Ramirez J, de La Bellacasa JP, Bauer T, Cabello H: Clinical diagnosis of ventilator associated pneumonia revisited: comparative validation using immediate post-mortem lung biopsies. Thorax 1999, 54: 867-873.
31. Andre C. Kalil, Mark L. Metersky, Michael Klompas, et al. Management of Adults With Hospital-acquired and Ventilator-associated Pneumonia: 2016 Clinical Practice Guidelines by the Infectious Diseases Society of America and the American Thoracic Society. Clin Infect Dis 2016; 63 (5): e61-e111.
32. Brun-Buisson C, Fartoukh M, Lechapt E, et al. Contribution of blinded, protected quantitative specimens to the diagnostic and therapeutic management of ventilator-associated pneumonia. Chest 2005; 128:533–44.
33. Raman K, Nailor MD, Nicolau DP, Aslanzadeh J, Nadeau M, Kuti JL. Early antibiotic discontinuation in patients with clinically suspected ventilator-associated pneumonia and negative quantitative bronchoscopy cultures. Crit Care Med 2013; 41:1656–63.

34. Singh N, Rogers P, Atwood CW, Wagener MM, Yu VL: Short-course empiric antibiotic therapy for patients with pulmonary infiltrates in the intensive care unit. A proposed solution for indiscriminate antibiotic prescription. Am J Respir Crit Care Med 2000, 162: 505-511.
35. Scott T. Micek; Suzanne Ward; Victoria J. Fraser. A Randomized Controlled Trial of an Antibiotic Discontinuation Policy for Clinically Suspected Ventilator Associated Pneumonia. Chest. 2004 May;125(5):1791-9.
36. Mandell LA, Wunderink RG, Anzueto A, et al; Infectious Diseases Society of America; American Thoracic Society. Infectious Diseases Society of America/American Thoracic Society consensus guidelines on the management of community-acquired pneumonia in adults. Clin Infect Dis. 2007;44(suppl 2):S27-S72.
37. Canadian Critical Care Trials Group: A randomized trial of diagnostic techniques for ventilator-associated pneumonia. N Engl J Med 2006, 355: 2619-2630.
38. Berton DC, Kalil AC, Cavalcanti M, Teixeira PJ: Quantitative versus qualitative cultures of respiratory secretions for clinical outcomes in patients with ventilator-associated pneumonia Cochrane Database Syst Rev CD006482. 2012.
39. Jones AE, Trzeciak S, Kline JA. The Sequential Organ Failure Assessment score for predicting outcome in patients with severe sepsis and evidence of hypoperfusion at the time of emergency department presentation. Critical care medicine. 2009;37(5):1649-1654.
40. Piaggio G, Elbourne DR, Pocock SJ, Evans SJW, Altman DG, CONSORT Group for the. Reporting of Noninferiority and Equivalence Randomized Trials. JAMA [Internet]. 2012 Dec 26 [cited 2018 Sep 26];308(24):2594. Available from: http://jama.jamanetwork.com/article.aspx?doi=10.1001/jama.2012.87802
41. Durrleman S, Simon R. Planning and monitoring of equivalence studies. Biometrics.1990;46:329-336.
42. Moi Lin Ling, Anucha Apisarnthanarak, Gilbert Madriaga; The Burden of Healthcare-Associated Infections in Southeast Asia: A Systematic Literature Review and Meta-analysis, Clin Infect Dis. 2015; 60 (11): 1690–1699.

Protocol version 4.0 dated 15 October 2019

17. APPENDIX A: STUDY FLOW CHART

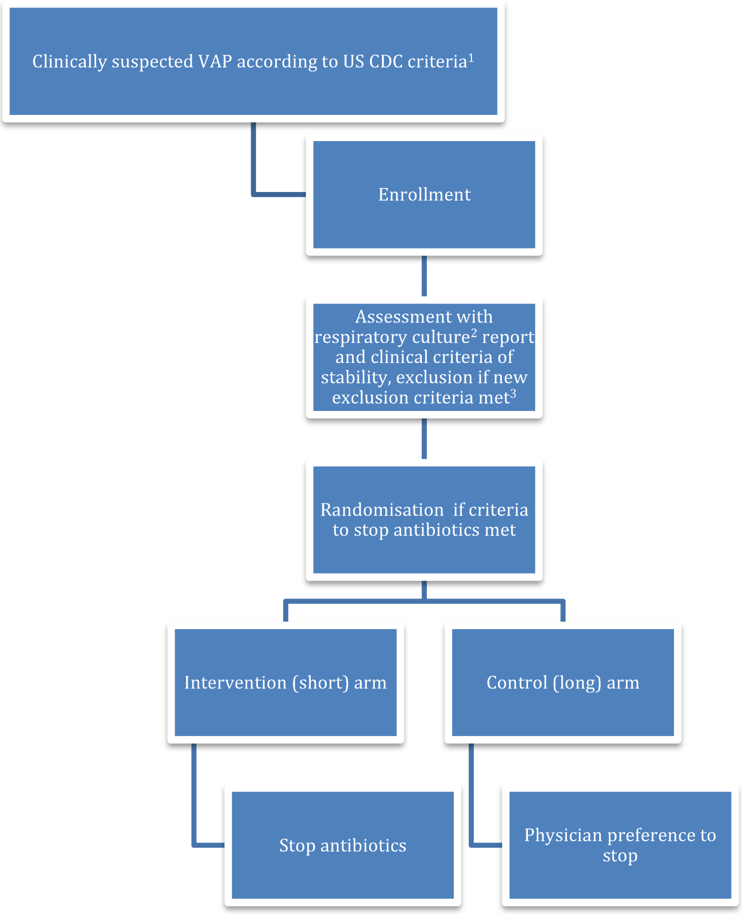

1. Patients who satisfy all the inclusion criteria below may be recruited:

a. Patients 18 years and older, and

b. Invasive mechanical ventilation ≥ 48 hours, and

c. Satisfy the US Centers for Disease Control and Prevention National Healthcare Safety Network VAP diagnostic criteria [29]

- At least one of the following:
  1. temperature > 38 °C
  2. white blood cell count ≥ 12,000 cells/mm^3 or ≤ 4,000 cells/mm^3
  3. altered mental status with no other causes in >70 year-olds; and
- Two or more chest imaging tests demonstrating at least one of the following:
  1. new and progressive OR progressive and persistent infiltrate
  2. new and persistent OR progressive and persistent consolidation
  3. new and persistent OR progressive and persistent cavitation, and
- At least two of the following:
  1. new onset of purulent sputum, or change in character of sputum, or increased respiratory secretions, or increased in suctioning requirements
  2. new onset or worsening tachypnea or dyspnea
  3. rales or bronchial breath sounds
  4. worsening gas exchange defined by oxygen desaturations (e.g., PaO2/FiO2 <240), increased oxygen requirements or increased ventilation demand

Mo Y, *et al. BMJ Open* 2021; 11:e050105. doi: 10.1136/bmjopen-2021-050105

2. Respiratory cultures can be quantitative or non-quantitative, invasive or non-invasive.
3. For those participants randomised to the intervention (short duration) arm, site investigators should assess the participants daily. Antibiotics should be stopped, if the criteria below are satisfied,

a. body temperature was ≤ 38.3°C (core body temperature measured orally or rectally) or 38.0°C (axillary) for 48 hours, and

b. hemodynamic instability (systolic blood pressure ≥ 90 mm Hg without inotropic support or no requirement of inotropic support to maintain systolic blood pressure above 90 mm Hg).

18. APPENDIX B: SCHEDULE OF STUDY PROCEDURES

**Procedures**	**Visit timing Day 0**	**Daily during hospitalisation**	**Weekly during hospitalisation**	**Day 28/60 (±5)**
	**Screening and enrolment**			**Follow up visit/ Last visit**
Informed consent	X			
Demographics	X			
Medical history and medical record review	X	X	X	X
Review antibiotics administration records	X	X	X	X
Physical examination	X			
Eligibility assessment	X			
Randomisation once clinical criteria for stability met		X		
Respiratory microbiology culture	X			
Stool and sputum sample for microbiome study	X		X	X
Review of antibiotics and stop antibiotics if criteria fulfilled		X		
Adverse event assessments (throughout the study)		X	X	X
