# Supplementary material for: Reducing antibiotic treatment duration for ventilator-associated pneumonia (REGARD-VAP): a trial protocol for a randomised clinical trial
Source: BMJ Open. 2021 May 13;11(5):e050105. doi: 10.1136/bmjopen-2021-050105 (PMC8126270; doi:10.1136/bmjopen-2021-050105)
Supplement: Supplementary data [file bmjopen-2021-050105supp002.pdf]

Supplementary file 2

Detailed definitions of primary and secondary outcomes.

	Definition	Method of data capture
Mortality	Death from any cause within 60 days of enrolment	Medical record review and/ or interview
Pneumonia recurrence	Additional episode of pneumonia as determined by two independent infectious disease or respiratory medicine experts blinded to the randomization within 60 days of enrolment	Medical record review for pneumonia symptoms following the performance of new respiratory culture or change in antibiotic regime by independent experts blinded to randomization
Ventilator-associated events	Additional episode of respiratory event fulfilling the US CDC NHSN criteria for VAP within 60 days of enrolment, but not determined to be pneumonia recurrence by two independent infectious disease or respiratory medicine experts blinded to the randomization	Medical record review for the US CDC NHSN criteria for VAP following the performance of new respiratory culture or change in antibiotic regime by independent experts blinded to the randomization
Duration of mechanical	Total number of days which a study participant is dependent	Medical record review

ventilation	on a ventilator for respiratory support within 60 days of enrolment	
Duration of hospitalization	Total number of days of hospitalization which the index episode of VAP that led to the study participant's enrolment took place	Medical record review
Duration of exposure to antibiotics during hospitalization	Total number of days which the study participant is administered antibiotics while inpatient for the index episode of VAP that led to the study participant's enrolment	Inpatient medicine prescription record review
Acquisition of multidrug resistant infection or colonization during hospitalization	Clinical cultures positive for third-generation cephalosporin resistant Enterobacteriaceae, carbapenem-resistant Gram-negative bacteria, methicillin-resistant Staphylococcus aureus and vancomycin-resistant *Enterococcus*	Review of all clinical cultures performed for the study participants during hospitalization
Number and types of extra-pulmonary	Clinical cultures positive for any organisms taken from normally sterile sites including	Review of all clinical cultures performed for the study participants during

infections identified from sterile sites during hospitalization	blood, cerebrospinal fluid, pleural fluid, peritoneal fluid, pericardial fluid, bone and synovial tissue, joint fluid, specimen obtained from surgery or aspirate from lymph node, brain, heart, liver, spleen, vitreous fluid, kidney, pancreas, ovary, vascular tissue and muscle	hospitalization

Mo Y, *et al. BMJ Open* 2021; 11:e050105. doi: 10.1136/bmjopen-2021-050105
