# Supplementary material for: Reducing antibiotic treatment duration for ventilator-associated pneumonia (REGARD-VAP): a trial protocol for a randomised clinical trial
Source: BMJ Open. 2021 May 13;11(5):e050105. doi: 10.1136/bmjopen-2021-050105 (PMC8126270; doi:10.1136/bmjopen-2021-050105)
Supplement: Reviewer comments [file bmjopen-2021-050105.reviewer_comments.pdf]

PEER REVIEW HISTORY

BMJ Open publishes all reviews undertaken for accepted manuscripts. Reviewers are asked to complete a checklist review form (**http://bmjopen.bmj.com/site/about/resources/checklist.pdf**) and are provided with free text boxes to elaborate on their assessment. These free text comments are reproduced below.

ARTICLE DETAILS

**TITLE (PROVISIONAL)**	Reducing Antibiotics Treatment Duration for Ventilator-associated Pneumonia (REGARD-VAP): A Trial Protocol for a Randomized Clinical Trial
**AUTHORS**	Mo, Yin; West, Timothy Eoin; Maclaren, Graeme; Booraphun, Suchart; Li, Andrew Yunkai; Kayastha, Gyan; Lau, Yie Hui; Chew, Yin Tze; Chetchotisakd, Ploenchan; TAMBYAH, Paul Anantharajah; Limmathurotsakul, Direk; Cooper, Ben

This article was not externally reviewed at BMJ Open. Protocols that have been independently assessed prior to submission to BMJ Open are usually fast-tracked to publication on the grounds that further substantial changes will not be possible. This independent assessment will usually be external, independent review for both a substantial grant award from a non-commercial or government funder and ethics approval.
